# Two New Aryltetralin Lignans from the Roots of *Dolomiaea souliei*

**DOI:** 10.3390/molecules17055544

**Published:** 2012-05-09

**Authors:** Hua Wei, Chunnian He, Yong Peng, Sen Zhang, Xiaoguang Chen, Peigen Xiao

**Affiliations:** 1 Institute of Medicinal Plant Development, Chinese Academy of Medical Science & Peking Union Medical College, Beijing 100193, China; 2 Key Laboratory of Bioactive Substances and Resources Utilization of Chinese Herbal Medicine, Ministry of Education, Peking Union Medical College, Beijing 100193, China; 3 Institute of Materia Medica, Chinese Academy of Medical Science & Peking Union Medical College, Beijing 100193, China

**Keywords:** Compositae, *Dolomiaea souliei*, aryltetralin, cytotoxicity

## Abstract

Two new aryltetralin-type lignans, dolomiaeasin A (**1**) and dolomiaeasin B (**2**), were isolated from the roots of *Dolomiaea souliei*. Their structures were elucidated by means of various spectroscopic analyses. The cytotoxicities of **1** and **2** were tested by the MTT method, and both compounds showed no significant cytotoxic activities against the A549 and A2780 human cancer cell lines. This is the first time that aryltetralin-type lignans were isolated from the genus *Dolomiaea*.

## 1. Introduction

*Dolomiaea souliei* (Franch.) Shih belongs to the *Dolomiaea* genus in the family Compositae, and is mainly distributed in western Sichuan and eastern Tibet [1]. *D. souliei* is a traditional Chinese medicine which is well known for its medicinal uses in relieving pain and different indigenous diseases [2]. Previous studies indicated that *D. souliei* is a rich source of sesquiterpenes, triterpenes and lignans, some of which have been reported to exhibit anti-tumor, anti-ulcer and anti-inflammatory activities [3,4]. In our search for biologically active compounds, we investigated the chemical constituents of this plant. In this study, two new aryltetralin-type lignans, dolomiaeasin A (**1**) and dolomiaeasin B (**2**), were isolated from the roots of *D. souliei*. Their structures were elucidated using UV, IR, 1D, 2D NMR and HR-ESI-MS experiments, while the configurations of both compounds were deduced by comparison of their CD data with those reported in the literature. This is the first report of aryltetralin-type lignans isolated from the genus *Dolomiaea*. Finally, the cytotoxicities of **1** and **2** were tested against the A549 and A2780 human cancer cell lines.

## 2. Results and Discussion

### 2.1. Structural Identification

Compound **1** was obtained as an amorphous powder, [*α*]
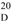
−4.0° (_*c*_ 0.225, MeOH). The HR-ESI-MS spectrum (*m/z* 391.13869 [M−H]^−^, calcd. for 391.13929) indicated the molecular formula of **1** to be C_20_H_24_O_8_. The ^1^H and ^13^C-NMR (APT) data of **1** showed the presence of a 1,3,4-trisubstituted benzene moiety [*δ*_H_: 6.86 (1H, s, H-2'), 6.76 (1H, m, H-5'), 6.78 (1H, m, H-6'); *δ*_C_: 134.0 (C-1'), 117.0 (C-2'), 148.7 (C-3'), 146.6 (C-4'), 115.5 (C-5'), 126.2 (C-6')], a 1,2,4,5-tetrasubstituted benzene moiety [*δ*_H_: 6.16 (1H, s, H-3), 6.66 (1H, s, H-6); *δ*_C_: 126.7 (C-1), 132.7 (C-2), 118.0 (C-3), 145.4 (C-4), 147.7 (C-5), 113.2 (C-6)], two methoxyl groups [*δ*_H_: 3.76 (3H, s, 3'-OCH_3_), 3.82 (3H, s, 5-OCH_3_); *δ*_C_: 56.6 (3'-OCH_3_), 56.7 (5-OCH_3_)] and other aliphatic signals [*δ*_H_: 4.38 (1H, s, H-7'), 3.49 (1H, d, *J* = 10.8 Hz, H-9'a), 3.59 (1H, d, *J* = 10.8 Hz, H-9'b), 2.56 (1H, d, *J* = 17.4 Hz, H-7a), 3.34 (1H, d, *J* = 17.4 Hz, H-7b), 3.85 (2H, m, H-9); *δ*_C_: 48.6 (C-7'), 65.0 (C-9'), 37.0 (C-7), 76.2 (C-8), 68.3 (C-9)]. The NMR signals were assigned with the aid of HSQC and HMBC spectra, and cross-peaks observed in the HMBC (H-2'/C-1', C-3', C-7'; 3'-OCH_3_/C-3'; H-5°/C-3', C-4'; H-6'/C-4'; H-7'/C-2', C-6', C-3; H-9'/C-7', C-8', C-8; H-3/C-7', C-2, C-4; 5-OCH_3_/C-5; H-6/C-1, C-5, C-7; H-7/C-8', C-6, C-8; H-9/C-8', C-7, C-8) indicated that **1** resembled the structure of (+)-cycloolivil [5]. 

**Figure 1:**
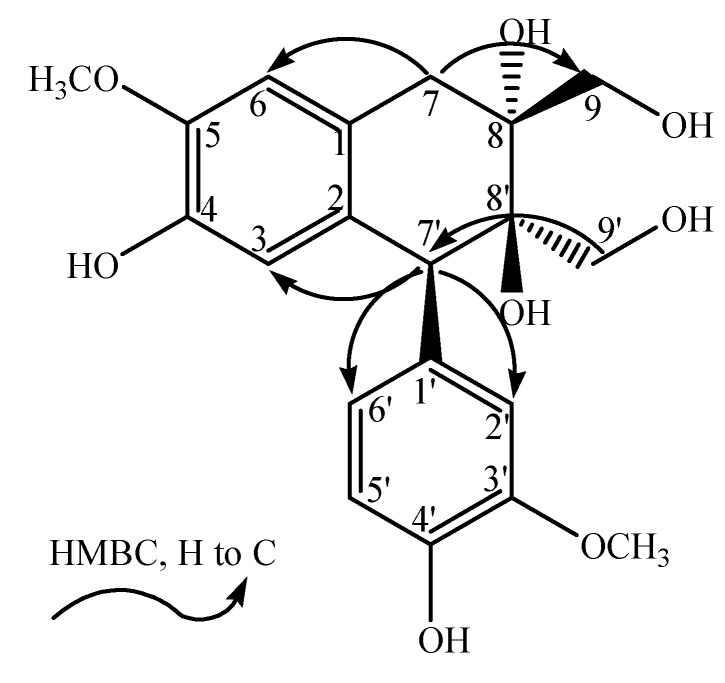
The key correlations of compound **1**.

The disappearance of H-8', sharp downﬁeld shift of C-8' (*δ*: 75.1) and obvious change of H-7' (a singlet) in **1** indicated that H-8' of (+)-cycloolivil was substituted by a group. When combined with HR-ESI-MS data, this group was inferred as a hydroxyl. A negative Cotton effect at 290 nm suggested that H-7' was *α* (*S* conﬁguration at C-7') [6]. The remaining chiral centers at C-8' and C-8 were assigned as 8'*R* and 8*R* configurations, for the CD data of **1** [(290 (−1.8), 271 (+0.5), 237 (+0.7)] being very similar to that of (+)-cycloolivil 6-*O*-*β*-D-glucoside which has the same chiral centers [7]. The results were in good accordance with the energy minimized conformation, which was obtained from a molecular modeling program in Discovery Studio 3.1. On basis of the above evidence, compound **1** was inferred as a structure with 7'*S*, 8'*R* and 8*R* configurations, and named dolomiaeasin A (Figure 1).

Compound **2** was obtained as an amorphous powder, [*α*]
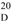
−16.3° (_*c*_ 0.24, MeOH). The HR-ESI-MS spectrum (*m/z* 391.13897 [M−H]^−^, calcd. for 391.13929) indicated the molecular formula of **2** to be C_20_H_24_O_8_. The NMR signals of **2** were assigned with the aid of HSQC and HMBC spectra and by comparison with the signals of **1**. The spectroscopic data of **2** suggested that it was another aryltetralin-type lignin, exhibiting an identical skeleton of **1**.

**Figure 2:**
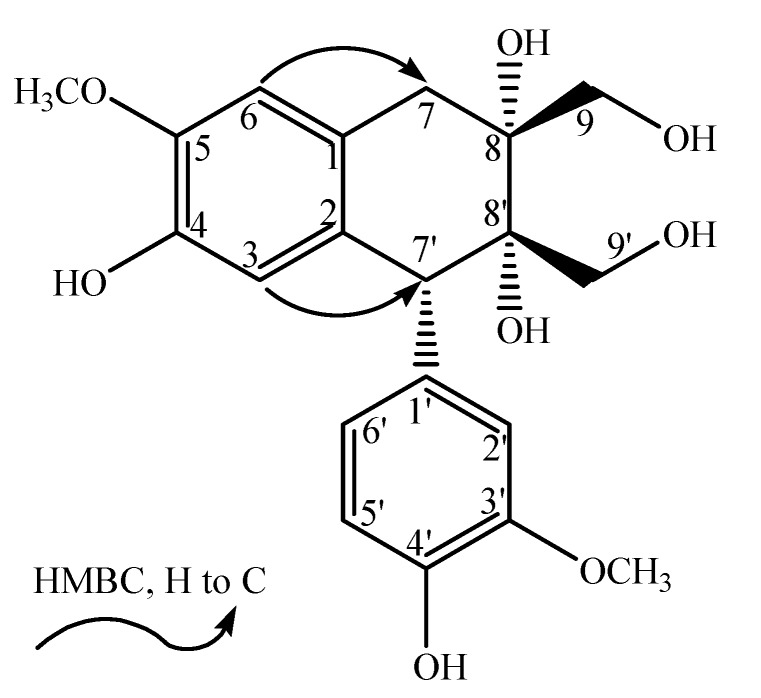
The key correlations of compound **2**.

Differences in chemical shift values and CD signals suggested a different stereochemistry of **2**. A positive Cotton effect at 291 nm revealed that H-7' was *β* (*R* conﬁguration at C-7') [6]. An opposite configuration of 7'-phenyl and 8'-CH_2_OH was inferred for there was no NOE correlation observed between H-9' and H-2'/H-6', *i.e*., the configuration at C-8' was 8'*S*. Differences in rotation values, CD and NMR revealed that these two compounds were not enantiomers. Thus, the remaining chiral centre at C-8 was inferred as 8*R*. On basis of the above deductions, the elucidation of compound **2** was characterized as 7'*R*, 8'*S* and 8*R*, and named dolomiaeasin B (Figure 2).

### 2.2. Cytotoxic Activity

While studies have indicated that an aryltetralin lactone (e.g., podophyllotoxin) and its derivatives were potent anticancer agents [8], compounds **1** and **2** showed no significant cytotoxic activities, with IC_50_ values exceeding 20 μM, when assessed against the A549 and A2780 human cancer cell lines. 

## 3. Experimental

### 3.1. General

Optical rotations were obtained on a Perkin-Elmer 341 digital polarimeter (Waltham, MA, USA). UV and IR spectra were recorded on Shimadzu UV2550 (Tokyo, Japan) and FTIR-8400S spectrometer (Tokyo, Japan), respectively. CD spectra were recorded on a JASCO J-815 spectropolarimeter (Tokyo, Japan). NMR spectra were obtained with a Bruker AV Ⅲ 600 NMR spectrometer (chemical shift values are presented as *δ* values with TMS as the internal standard; Munich, German). HR-ESI-MS spectra were performed on a LTQ-Obitrap XL spectrometer and HPLC on a Shimadzu system (Agilent Eclipse XDB-18, 5 μm, 9.4 × 250 mm; detection: UV at 210 nm; Santa Clara, CA, USA). ODS gel (50 µm, YMC, Kyoto, Japan), Sephadex LH-20 (Pharmacia, Stockholm, Sweden) and silica gel (100–200 and 300–400 mesh, Qingdao Marine Chemical Plant, Qingdao, China) were used for column chromatography. Precoated silica gel GF_254_ plates were used for TLC (Qingdao Marine Chemical Plant, Qingdao, China).

### 3.2. Plant Material

The roots of *D. souliei* were collected from Sichuan province, China, in September 2010. A voucher specimen (No. 20100810wh1) was deposited in the herbarium of Institute of Medicinal Plant Development, Chinese Academy of Medical Science & Peking Union Medical College, Beijing, China.

### 3.3. Extraction and Isolation

The air-dried roots of *D. souliei* (12.0 kg) were extracted with 70% ethanol (3 × 50 L, 3 h) at room temperature. After removing the solvent, the ethanol extract was suspended in distilled water and successively partitioned with petroleum ether, CHCl_3_, EtOAc and *n*-BuOH. The EtOAc fraction (63.0 g) was subjected to silica gel (100−200 mesh) column chromatography eluted with a solvent system of CHCl_3_-MeOH (100: 2–100: 33) to give 11 fractions. Fraction 4 was successively subjected to column chromatography over ODS gel (50 *µ*m), silica gel (300−400 mesh), Sephadex LH-20 and HPLC (H_2_O: MeOH = 90: 10−40: 60) to afford compound **1** (9 mg) and **2** (6 mg).

### 3.4. Spectral Data

*Dolomiaeasin A* (**1**): HR-ESI-MS spectrum (*m/z* 391.13869 [M−H]^−^, calcd. for C_20_H_23_O_8_, 391.13929); [*α*]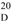: −4.0° (*c* 0.225, MeOH); UV *λ*_max_ (log *ε*) nm (MeOH): 207 (4.46), 283 (3.60); CD nm (*Δε*) (*c* 1.28 × 10^−3^ mol/L, MeOH): 290 (−1.8), 271 (+0.5), 237 (+0.7); IR *ν*_max_ cm^−1^ (KBr): 3392, 2928, 1647, 1516, 1445, 1383, 1261, 1126, 1100, 1033, 798, 762, 652, 601; ^1^H-NMR (CD_3_OD, 600 MHz) *δ*: 6.86 (1H, s, H-2'), 3.76 (3H, s, 3'-OCH_3_), 6.76 (1H, m, H-5'), 6.78 (1H, m, H-6'), 4.38 (1H, s, H-7'), 3.49 (1H, d, *J* = 10.8 Hz, H-9'a), 3.59 (1H, d, *J* = 10.8 Hz, H-9'b), 6.16 (1H, s, H-3), 3.82 (3H, s, 5-OCH_3_), 6.66 (1H, s, H-6), 2.56 (1H, d, *J* = 17.4 Hz, H-7a), 3.34 (1H, d, *J* = 17.4 Hz, H-7b), 3.85 (2H, m, H-9); ^13^C-NMR (CD_3_OD, 150 MHz) *δ*: 134.0 (C-1'), 117.0 (C-2'), 148.7 (C-3'), 56.6 (3'-OCH_3_), 146.6 (C-4'), 115.5 (C-5'), 126.2 (C-6'), 48.6 (C-7'), 75.1 (C-8'), 65.0 (C-9'), 126.7 (C-1), 132.7 (C-2), 118.0 (C-3), 145.4 (C-4), 147.7 (C-5), 56.7 (5-OCH_3_), 113.2 (C-6), 37.0 (C-7), 76.2 (C-8), 68.3 (C-9).

*Dolomiaeasin B* (**2**): HR-ESI-MS spectrum (*m/z* 391.13897 [M−H]^−^, calcd. for C_20_H_23_O_8_, 391.13929); [*α*]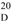: −16.3° (*c* 0.24, MeOH); UV *λ*_max_ (log *ε*) nm (MeOH): 210 (4.8), 284 (3.99); CD nm (*Δε*) (*c* 2.55 × 10^−3^ mol/l, MeOH): 291 (+3.1), 273 (−1.2), 230 (+1.9); IR *ν*_max_ cm^−1^ (KBr): 3419, 2954, 1652, 1520, 1456, 1373, 1260, 1127, 1097, 1033, 803, 773, 645, 597; ^1^H-NMR (CD_3_OD, 600 MHz) *δ*: 6.78 (1H, s, H-2'), 3.78 (3H, s, 3'-OCH_3_), 6.72 (1H, d, *J* = 8.4 Hz, H-5'), 6.63 (1H, m, H-6'), 4.06 (1H, s, H-7'), 3.55 (1H, d, *J* = 10.2 Hz, H-9'a), 3.96 (1H, d, *J* = 10.2 Hz, H-9'b), 6.30 (1H, s, H-3), 3.83 (3H, s, 5-OCH_3_), 6.67 (1H, s, H-6), 3.02 (2H, m, H-7), 3.34 (1H, d, *J* = 11.4 Hz, H-9a), 3.91 (1H, d, *J* = 11.4 Hz, H-9b); ^13^C-NMR (CD_3_OD, 150 MHz) *δ*: 133.3 (C-1'), 117.0 (C-2'), 148.4 (C-3'), 56.6 (3'-OCH_3_), 146.7 (C-4'), 115.5 (C-5'), 125.7 (C-6'), 56.3 (C-7'), 77.1 (C-8'), 67.0 (C-9'), 127.5 (C-1), 131.4 (C-2), 117.6 (C-3), 145.8 (C-4), 148.2 (C-5), 56.6 (5-OCH_3_), 112.9 (C-6), 39.2 (C-7), 77.6 (C-8), 67.7 (C-9).

### 3.5. Bioassays

Compounds **1** and **2** were assessed by the MTT method using the A549 and A2780 human cancer cell lines. Cells were seeded in 96-well plates and incubated at 37 °C, 5% CO_2_ for 24 h. Then 150 μL of five different concentrations (0.2, 0.5, 1, 2, 5, 10 μM) for each compound (dissolved in DMSO) were added to each well and incubated for another 24 h. After removing the supernatant, 150 μL of MTT (0.5 mg/mL) were added to each well and incubated for 4 h. Finally, the liquid in the wells was removed, DMSO (150 μL) was added, and the absorbance at 570 nm was recorded on a microplate reader (Wellscan MK3, Labsystems Dragon, Helsinki, Finland).

## 4. Conclusions

Two new aryltetralin-type lignans, dolomiaeasin A (**1**) and dolomiaeasin B (**2**), were isolated from the roots of *Dolomiaea souliei*. Both compounds showed no significant cytotoxicities against the A549 and A2780 human cancer cell lines. To the best of the authors’ knowledge, this is the first report of aryltetralin-type lignans from the genus *Dolomiaea*.
